# *Meloidogyne graminicola* protein disulfide isomerase may be a nematode effector and is involved in protection against oxidative damage

**DOI:** 10.1038/s41598-019-48474-w

**Published:** 2019-08-16

**Authors:** Zhong-ling Tian, Ze-hua Wang, Munawar Maria, Nan Qu, Jing-wu Zheng

**Affiliations:** 1 Laboratory of Plant Nematology, Institute of Biotechnology, College of Agriculture and Biotechnology, Zhejiang University, Hangzhou, 310058 Zhejiang P.R. China; 2 Institute of Insect Science, College of Agriculture and Biotechnology, Zhejiang University, Hangzhou, 310058 Zhejiang P.R. China; 3 Key Lab of Molecular Biology of Crop Pathogens and Insects, Ministry of Agriculture, Hangzhou, 310058 P.R. China

**Keywords:** RNAi, Parasite host response, Protein purification

## Abstract

The rice root-knot nematode, *Meloidogyne graminicola*, is a serious pest in most rice-growing countries. Usually, nematodes employ antioxidants to counteract the harm of reactive oxygen species (ROS) and facilitate their infection. Here the gene encoding *M*. *graminicola* protein disulphide isomerase (*MgPDI*) was identified. The deduced protein is highly conserved in the putative active-site Cys-Gly-His-Cys. *In situ* hybridization showed that *MgPDI* was specifically localized within esophageal glands of pre-parasitic second stage juveniles (J2s). *MgPDI* was significantly up-regulated in the late parasitic J2s. Characterization of the recombinant protein showed that the purified MgPDI exhibited similar activities to other oxidases/isomerases such as the refolding of the scrambled RNase and insulin disulfide reductase and the protection of plasmid DNA and living cells from ROS damage. In addition, silencing of *MgPDI* by RNA interference in the pre-parasitic J2s lowered their multiplication factor. *MgPDI* expression was up-regulated in the presence of exogenous H_2_O_2_, whereas *MgPDI* silencing resulted in an increase in mortality under H_2_O_2_ stress. MgPDI is localized in the apoplast when transient expression in *Nicotiana benthamiana* leaves. The results indicated that MgPDI plays important roles in the reproduction and pathogenicity of *M*. *graminicola* and it also contributes to protecting nematodes from exogenous H_2_O_2_ stress.

## Introduction

Rice (*Oryza sativa*) is an important cereal crop; more than half of the world population relies on rice. Among rice pathogens, rice root-knot nematode, *Meloidogyne graminicola* (Mg), is considered to be a quarantine pest in most of the rice-growing countries and cause significant yield reductions^1–3^. *Meloidogyne graminicola* is an obligate sedentary endoparasite of rice^4^. The second stage juvenile (J2) penetrates the epidermal root tip and migrates intercellularly along the vascular zone and becomes sedentary to induce and establish the feeding site. Then the J2 develops into a third (J3) and then fourth (J4) stage juvenile and eventually into an adult where the male moves out from the root while the female remains embedded within the root^5^. Individuals of Mg can produce many generations within a single rice growing season under favorable conditions^6^.

Reactive oxygen species (ROS) can be involved in host defense reactions through the oxidation of phenolic compounds into more toxic quinones and lignin-like compounds. Plants generate ROS as signaling molecules to control various processes including pathogen defense and programmed cell death under biotic stresses^7^. However, in order to survive, aerobic cells may reduce ROS-related oxidative stress by expressing a set of antioxidant enzymes, including thioredoxin (Trx)^8^.

The protein disulphide isomerase (PDI) family, a member of the thioredoxin superfamily, can catalyze the formation, reduction, and isomerization of disulfide bonds of proteins^9^. Protein disulphide isomerase includes two catalytically active thioredoxin domains containing characteristic Cys-Gly-His-Cys (CGHC) active-site motifs, namely domains a and a’ which are separated by two homologous but inactive internal domains b and b’^10,11^. Protein disulfide isomerase is a multifunctional enzyme because it can catalyze thiol oxidation reactions, disulfide reduction, and isomerisation *in vitro* under different redox conditions. Previous research has demonstrated that PDI participates in many biological processes by regulating disulfide bonds in different substrates. For example, PDI can release the cholera toxin active chain A from the endoplasmic reticulum to the cytosol of an infected cell and permit HIV to enter into host cells with infectious diseases^12^. The PDI from *Caenorhabditis elegans* was reported to play a role in the formation of the cuticle^13^. While HsPDI plays a role probably by inducing local changes in the redox status of infected host tissue^14^. Other studies have reported that PDIs of pathogens also play important roles in virulence when infecting a host^15^. Over-expression of *Leishmania major* PDI (LmPDI) can promote the virulence of parasitic strains of *Leishmania* species to cause leishmaniasis^16^. However, PDI inhibitors can hinder parasite growth in culture^9^. Expression of *PDI* from the oomycete plant parasite *Phytophthora parasitica* induces strong cell death in *Nicotiana benthamiana* leaves while the effect is reduced in a gene mutant^17^. Researchers have studied PDI in a number of organisms and report that PDI is evolutionarily conserved from prokaryotes to eukaryotes^9^. Characterizing PDI from these organisms can contribute to our understanding of redox control and provide information on novel targets for pathogen control. However, little is known about the PDI family from *M*. *graminicola*.

In the present study, a 56-kDa protein designated MgPDI that contains a CGHC active motif in the rice root-knot nematode, *M*. *graminicola*, was identified and characterized. It is localized in the apoplast when transient expression in *N*. *benthamiana* leaves. The enzymatic activities of MgPDI include oxidation, isomerisation, and reduction of substrates. MgPDI likely plays an important role in protecting *M*. *graminicola* against rice-released ROS and facilitating *M*. *graminicola* infestation of rice hosts and its role was demonstrated in this study.

## Results

### Cloning and sequence analysis of *MgPDI*

**Figure 1:**
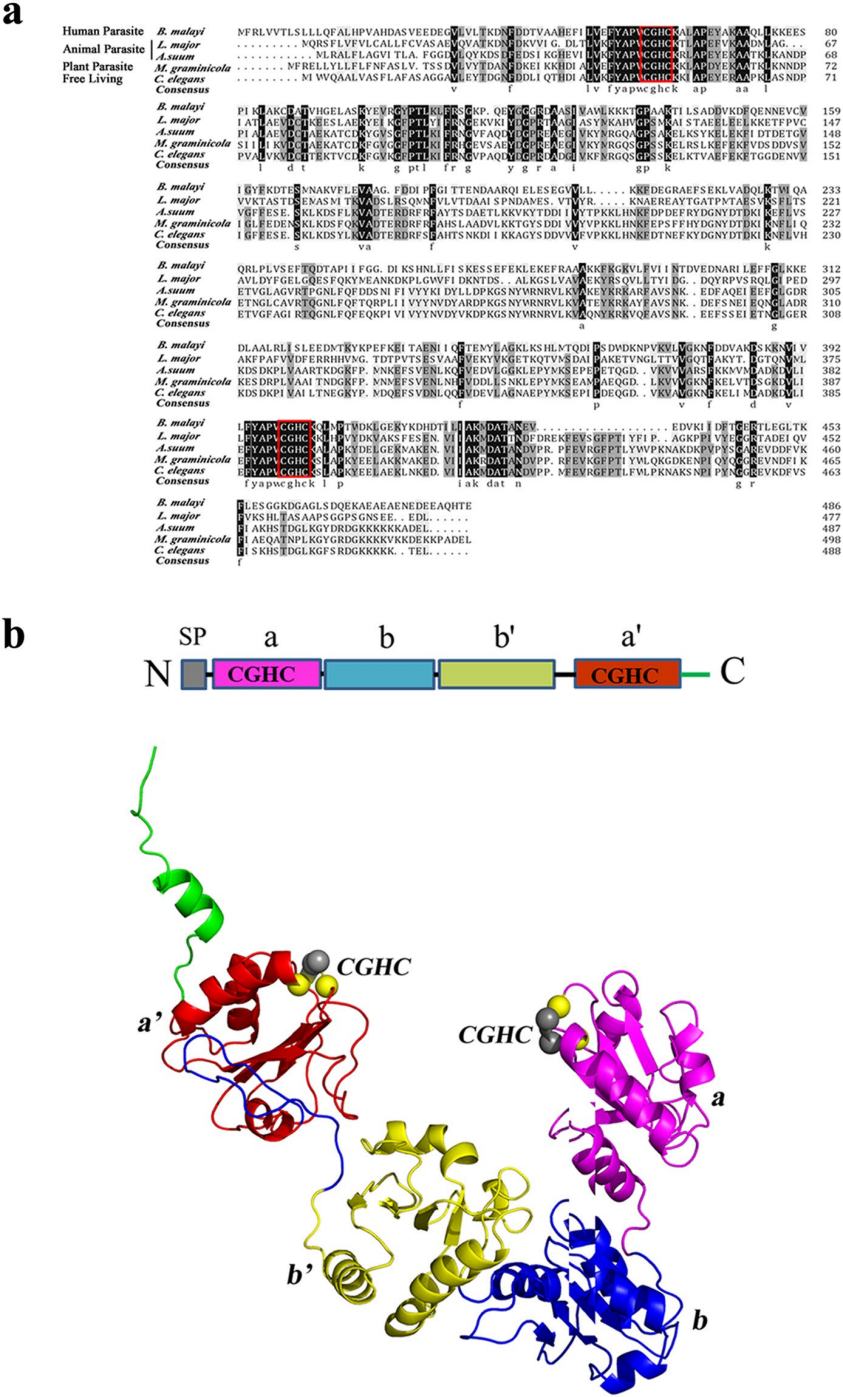
Sequence and structural analysis of MgPDI. (**a**) Alignment of MgPDI with orthologues in other nematode species. (**b**) Schematic drawing of MgPDI primary and tertiary structures.

The full-length *MgPDI* was identified through RACE-PCR and was deposited in GenBank under accession number **MH392200**. The ORF of the *MgPDI* was from nucleotide 170–1666, encoding a putative protein of 498 amino acids with a deduced signal peptide cleavage site between residues 20 and 21. The predicted isoelectric point and molecular weights were 7.02 and 56.96 kDa, respectively. Further domain analysis revealed that the putative protein contained four conserved thioredoxin domains (a, b, b’, a’) with two catalytic domains (CGHC) (Fig. 1a,b). Protein disulphide isomerase has been found in humans and other animals and plant-parasitic and free-living nematodes. Results of a BLASTP search showed that MgPDI shared 79%, 71%, 69%, 32% and 33% identity to PDI from *Heterodera schachtii*, *Caenorhabditis elegans* [NP_491995], *Ascaris suum* [ERG84937], *Leishmania major* [AAN75008], and *Brugia malayi* [XP_001899304], respectively.

In their tertiary structure, the four domains are found to be spatially organized in the shape of a twisted “U”, in which the a and a’ domains are located at the ends while the b and b’ domains formed the base of the “U”; Four conserved thioredoxin domains (a, b, b′, a′) are highlighted in purple, blue, yellow and red, respectively; The C-terminal extension is in green; Two catalytic domains (CGHC) are highlighted in the MgPDI tertiary structure as colored spheres. One sphere represents one of the atoms in the corresponding amino acids (Fig. 1b).

### *In situ* hybridization and expression analysis

**Figure 2:**
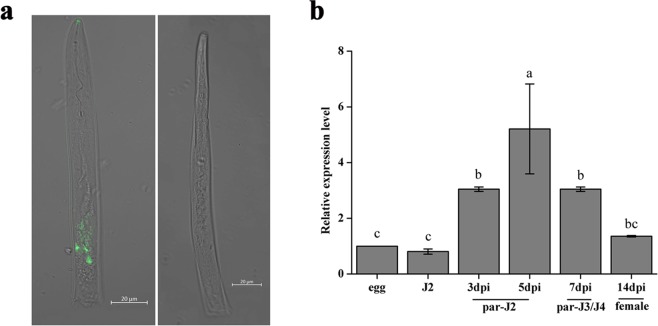
Expression patterns of *MgPDI* in *M*. *graminicola*. (**a**) Localization of *MgPDI* by *in situ* hybridization. Right: antisense cDNA probes; left: sense: cDNA probes. (**b**) Expression pattern of *MgPDI* in five different life stages of *M*. *graminicola*. Vertical bars represent the means ± SD.

*In situ* hybridization was performed to localize the expression of *MgPDI* in the pre-parasitic juveniles of *M*. *graminicola*. The DIG-labelled antisense probe of *MgPDI* specifically hybridized with transcripts in the subventral gland cells of the pre-parasitic J2s (pre- J2s), while no hybridization was observed in the negative control, the labeled sense probe (Fig. 2a). To further investigate the expression pattern of *MgPDI* during different developmental stages of *M*. *graminicola*, we used cDNA generated from nematode RNA isolated at different pre-parasitic (eggs and freshly hatched J2s) and parasitic developmental stages (par-J2s, par-J3s/par-J4s and young females) in qRT-PCR analyses. The expression level of *MgPDI* increased during the sedentary stages of nematode development, reaching its maximum in the late parasitic J2s (5 days post-infection, 5dpi) with a 6-fold increase compared with pre-parasitic J2s. In J3/J4s and young females, expression decreased but was still higher when compared with unhatched J2s in eggs and hatched pre-parasitic J2s (Fig. 2b).

### Recombinant MgPDI: optimization and validation

**Figure 3:**
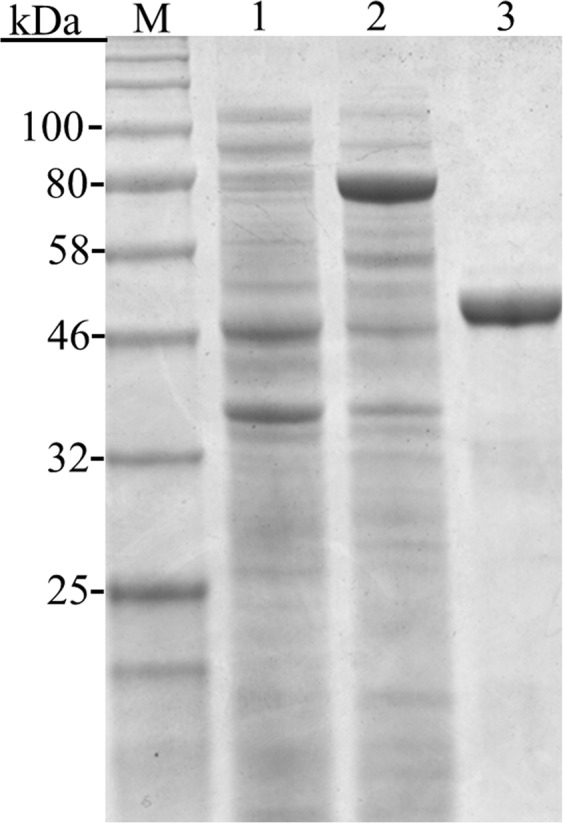
The 12% SDS-PAGE analysis of MgPDI expressed in *E*. *coli* BL21 (DE3). The total proteins (lane 1), the expressed product of pET-32a-MgPDI induced by IPTG (lane 2) and enriched nontagged MgPDI (lane 3) were run on one gel.

We expressed MgPDI in *E*. *coli* BL21 (DE3) cells (Supplementary Fig. S1). Isopropyl-B-D-thiogalactoside induced the *E*. *coli* expression of the recombinant protein (Fig. 3, lane 2). To exclude the possible effects of the tags on the activity of MgPDI, thrombin was used to cleave N-terminal fusion tags and the recombinant protein yielded an enriched nontagged protein band after purification (Fig. 3, lane 3). The final concentration of MgPDI was 2 mg/ml after removing the thrombin. Mass spectrometry analysis was used to validate the identity of the expressed protein. The purified recombinant protein exhibited high sequence homology with the peptide fragments deduced from *MgPDI* where the score was 11512.77% and coverage was 72.09% (Supplementary Table S1).

### Oxidoreductase activity of MgPDI

**Figure 4:**
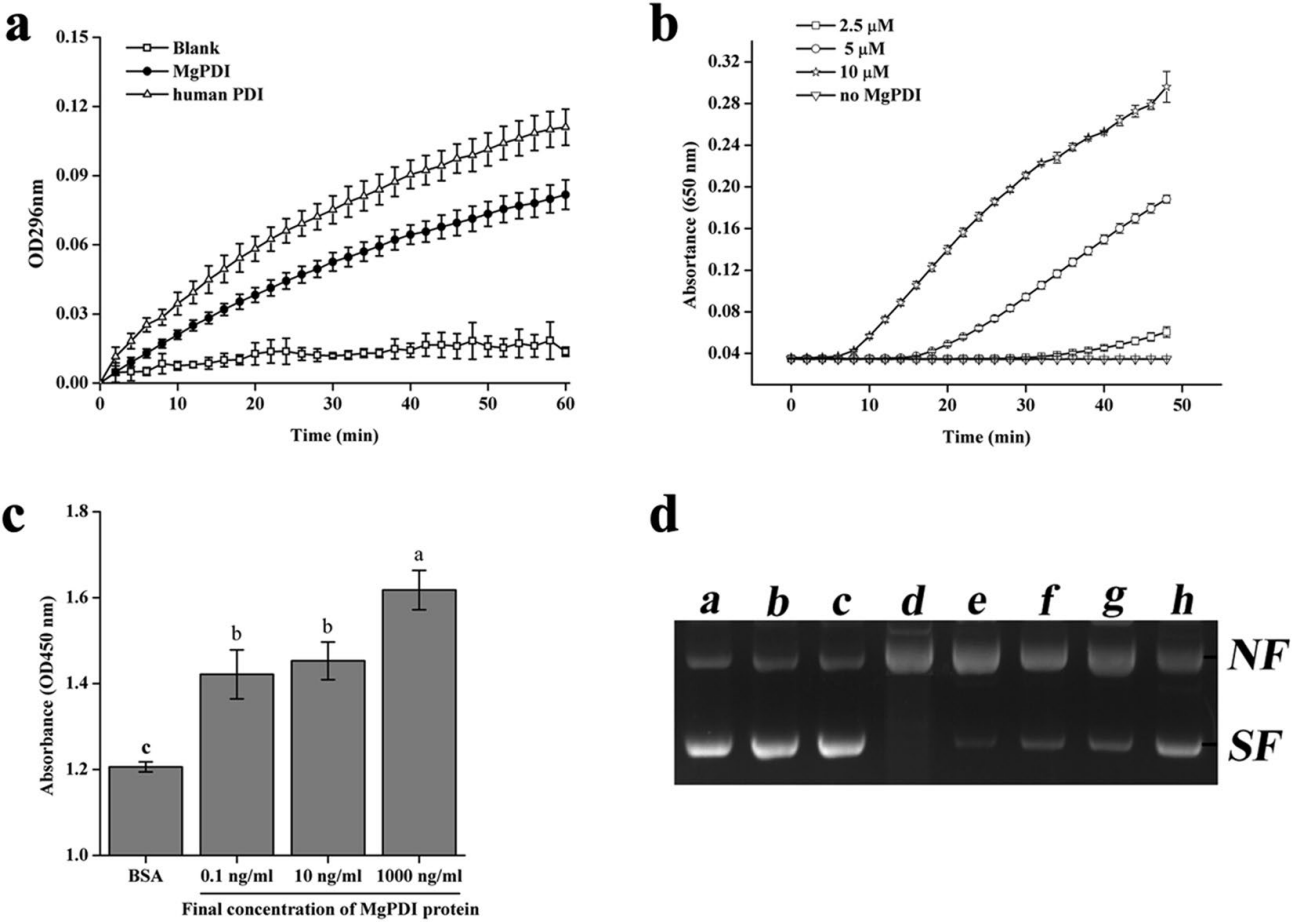
Biological activity of recombinant MgPDI. (**a**) Thiol isomerase activity of the purified recombinant MgPDI. (**b**) Dithiothreitol-mediated insulin reduction by recombinant MgPDI. (**c**) MgPDI protects H_2_O_2_-stressed HEK293 cells. (**d**) Potential of recombinant MgPDI to protect super-coiled DNA from cleavage in an MFO system. Lanes: a, pGEM-T without any treatment; b, pGEM-T incubated with 1.65 mM DTT; c, pGEM-T incubated with 16.5 mM FeCl3; d, pGEM-T incubated with MFO system; e–h, pGEM-T incubated with MFO system and different concentrations of purified MgPDI. NF, nicked form; SF, super-coiled form of pGEM-T. Vertical bars represent the means ± SD.

Oxidase/isomerase activity was analyzed using an RNase A renaturation assay, and the results indicated that MgPDI exhibited the properties of oxidative folding on RNase A, similar to the properties of human PDI. The activity of MgPDI induced a time-dependent increase in denatured RNase A refolding; the velocity of cCMP hydrolysis catalyzed by renatured RNase A increased with the time allowed for the refolding reaction (Fig. 4a). The human PDI used as a positive control showed marginally greater oxidase/isomerase activity than the MgPDI in the present study. For the MgPDI reductive assay, the decline in bovine insulin disulfide bonds with the increase in MgPDI concentrations was detected by the increasing turbidity in the reactions. The negative control reaction without MgPDI, however, showed no insulin disulphide bond reduction (Fig. 4b).

### MgPDI function as antioxidants

The influence of MgPDI on viability of HEK293 cells under H_2_O_2_ stress was determined using a cell proliferation assay kit. Overall, viability of cells in the MgPDI-treated groups was higher than that of the BSA-treated control when exposed to the H_2_O_2_ stress. Moreover, viability increased with the increase of MgPDI concentration (Fig. 4c). The degree of DNA damage was indicated by distinct mobility-shift patterns produced by the two DNA bands after electrophoretic resolution on a gel; MgPDI appears to rescue the plasmid DNA-nicking reaction in a dose-dependent manner (Fig. 4d, lanes e to h). However, the DNA underwent maximum damage in the MFO system when MgPDI was absent (Fig. 4d, lane d).

### Hydrogen peroxide content determination

**Figure 5:**
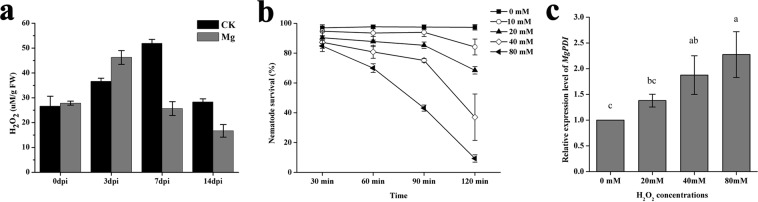
*MgPDI* expression is triggered by H_2_O_2_ and increases H_2_O_2_ tolerance. (**a**) The H_2_O_2_ content per gram of fresh root tissue. (**b**) Mortality rate of freshly hatched J2s exposed to H_2_O_2_. (**c**) The relative mRNA expression levels of *MgPDI* in response to the H_2_O_2_ stress. Vertical bars represent the means ± SD.

The hydrogen peroxide (H_2_O_2_) level was determined in rice roots, and increased level of H_2_O_2_ was found at 3 dpi, however, H_2_O_2_ levels in knots were lower at 7 dpi and 14 dpi compared with the root tips without Mg infection (Supplementary Fig. S2, Fig. 5a).

### *MgPDI* expression is induced by H_2_O_2_ and increases H_2_O_2_ tolerance

In this study we observed that J2s can survive up to 90 min in 10 mM H_2_O_2_ without a significant increase in mortality rate; however, overall mortality of J2s noticeably increased with increasing H_2_O_2_ concentrations (Fig. 5b). Next, qRT-PCR was used to analyse the expression of *MgPDI* in response to the H_2_O_2_ stress. A significant increase in transcript abundance of *MgPDI* was observed when comparing juveniles that were exposed to H_2_O_2_ (20 mM–80 mM) for 30 min with J2s that were treated with water (Fig. 5c).

### Influence of RNAi silencing of MgPDI on nematode parasitism

**Figure 6:**
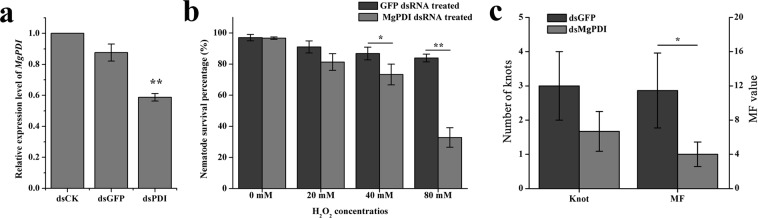
Effect of *MgPDI* expression on rice susceptibility to *M*. *graminicola* infection. (**a**) *MgPDI* expression in nematode after treatment with dsRNA. Asterisks indicate significant differences based on Tukey’s test (*P < 0.05; **P < 0.01). (**b**) Effect of *MgPDI* silencing on H_2_O_2_ stress tolerance. (**c**) Average numbers of knots and the MF values at 15 dpi. Vertical bars represent the means ± SD.

*In vitro* RNAi targeting of *MgPDI* was performed to analyze whether *MgPDI* plays an important role in parasitism. The results of qRT-PCR experiments showed a decrease in the transcript abundance of *MgPDI* when the nematodes were soaked with dsRNA against *MgPDI*, indicating the effective silencing of *MgPDI* (Fig. 6a). We examined the mortality rates of *MgPDI* or *GFP* dsRNA-treated J2s after soaking them in various concentrations of H_2_O_2_ like those used in the transcript expression experiment. A significantly lower percentage in survival of *MgPDI* dsRNA-treated J2s was observed than of *GFP* dsRNA-treated J2s where both groups were exposed to H_2_O_2_ (Fig. 6b).

The infection studies showed that the treatment of J2 with *MgPDI* dsRNA significantly reduced the reproductive ability of nematodes (MF = 6.22) compared to that of the treatment of J2 with *GFP* dsRNA (MF = 11.46) (Fig. 6c).

### MgPDI is localised in the plant apoplastic space

**Figure 7:**
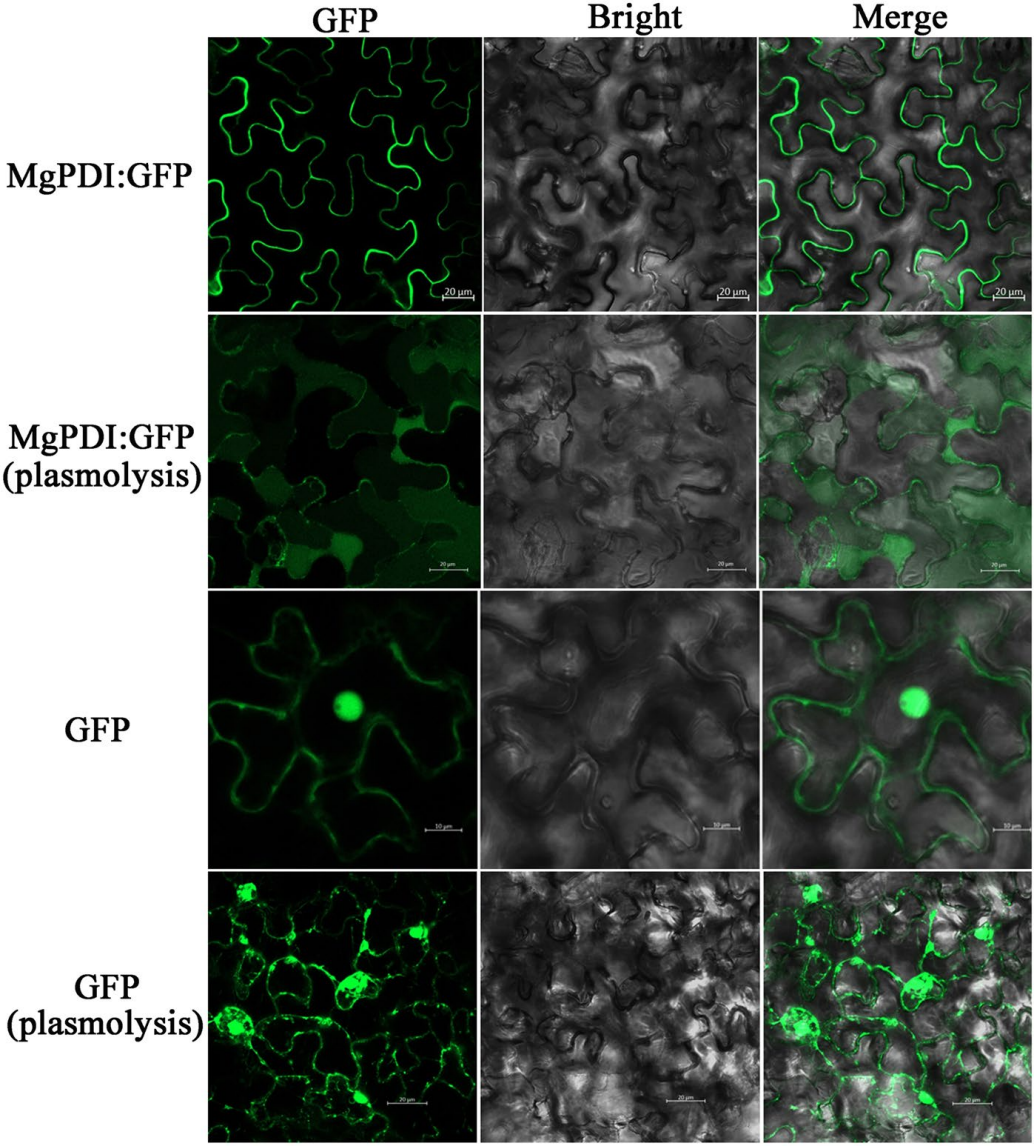
Subcellular localization of transiently expressed MgPDI:GFP fusions in *N*. *benthamiana* leaves. The first and third panel were MgPDI:GFP/GFP in normal cell, and the second and forth panel was MgPDI:GFP/GFP in plasmolysed cell.

To investigate the subcellular localization of MgPDI in plant cells, the reporter protein GFP was fused to the C-terminus of the MgPDI protein. The GFP signal from the MgPDI:GFP fusion expressed in *N*. *benthamiana* was observed at the cell periphery. However, the GFP signal was observed in the apoplastic space when the plant cells were plasmolysed by treating with 1 M NaCl_2_ which indicated that the localization of MgPDI was in the apoplast (Fig. 7).

## Discussion

The present study identifies *MgPDI* encoding a typical PDI in the endoparasitic nematode *M*. *graminicola*, and MgPDI contains a 20-amino acid signal peptide at its N-terminal and two catalytic thioredoxin-like domains (active sites), each containing the canonical CGHC motif, and two non-catalytic domains (Fig. 1a,b). PDI is specifically responsible for folding proteins into the endoplasmic reticulum^18^. In addition, due to the active motifs in PpPDI1, its enzymatic function purportedly causes cell death in *P*. *parasitica*^17^. Our results also showed that the active catalytic motifs are highly conserved in the sequences under investigation (Fig. 1a). The described sequence was annotated as MgPDI for its arrangement of PDI classical domains.

*In situ* hybridization showed that *MgPDI* was expressed in oesophageal gland cells of *M*. *graminicola* pre-parasitic J2s, which is said to be nematode secretory effector proteins origin and presumed to be involved in the early parasitic stages of Mg. In addition, the expression analysis in different life stages of *MgPDI* found that transcription of *MgPDI* was up-regulated during early parasitic stages and peaked at 5 dpi, which is similar with the expression pattern of *HsPdi* in *H*. *schachtii*^14^.

To explore other functional characteristics of MgPDI, a large quantity of active recombinant MgPDI was obtained by induction at optimal conditions (20 °C overnight with 0.5 mM IPTG). Previous studies demonstrated that the enzymatic activity of PDI lies in its roles in oxidization, isomerization, and reduction^9^. In addition, the oxidase/isomerase activity of MgPDI utilizes the refolding of scrambled RNase^19^. A classical assay of insulin disulfide bridge-reduction in the presence of DTT was performed to analyze the enzymatic activity of PDI reduction, and the results indicated that MgPDI catalysed the reduction of insulin disulfide bonds, as seen in previous studies^9^. Protein disulphide isomerase is a member of the thioredoxin superfamily, which is composed of several redox proteins that play key roles in many essential antioxidant and redox-regulatory processes^20,21^. As for the enzymatic activity of antioxidants, first, our results indicated that MgPDI functioned in HEK293 cells to reduce oxidative stress by H_2_O_2_, and in particular, the recombinant protein exerted its protective influence in a dose-dependent manner (Fig. 4c). To further confirm the ability of MgPDI to function as an antioxidant, the plasmid DNA nicking assay was performed to demonstrate the ability of MgPDI to protect super-coiled DNA from nicking. Results indicated that MgPDI (at 0.1, 1, 10 and 100 μg/ml) effectively protects the super-coiled DNA from damage (Fig. 4d). DNA protection against oxidative damage by thioredoxin has also been demonstrated in *Haemonchus contortus*^22^. This is the first report that a functional recombinant PDI protein was obtained from the plant parasitic nematode *M*. *graminicola* and confirmed that MgPDI encodes a functional protein disulfide isomerase.

To determine the importance of *MgPDI* in the parasitism of *M*. *graminicola*, *MgPDI* expression was knocked down using RNAi, which has been effectively used to research both sedentary and migratory endoparasites, such as plant-parasitic nematodes and the function of their pathogenicity factors^23^. Knocking down the expression of *MgPDI* in preparastitic nematodes reduced the MF by more than 45% compared with the MF when in the non-specific presence of dsRNA (GFP). Our results are in accordance with Habash *et al*., who reported that RNAi silencing of the *HsPDI* gene of *H*. *schachtii* resulted in a significant reduction in the number of eggs produced^14^. These findings indicated that protein disulfide isomerase is essential in the reproduction of *M*. *graminicola*.

Studies have shown that ROS causes damage to cellular organelles and inhibits cell functions by oxidizing DNA, proteins, and lipids^24^. However, plant-parasitic nematodes can encode various antioxidant enzymes including superoxide dismutase (SOD), catalase, peroxiredoxins, glutathione peroxidases and protein disulfide isomerase to protect against ROS^14,25–28^. Results of the present study support that the primary role of *MgPDI* is to protect Mg from damage by ROS. Previous research reported that the production of ROS during the oxidative burst reflected the defense response in plants^29^. In order to test whether the reactive oxygen species of rice infected with Mg were affected. The H_2_O_2_ level was determined in rice roots, and we found that H_2_O_2_ levels in knots were lower at 7 dpi and 14 dpi compared with the root tips without Mg infection. What’s more, we observed a significant increase in *MgPDI* expression in response to the exogenous stress of H_2_O_2_. In addition, the tolerance of *MgPDI*-silenced nematodes to different concentrations of H_2_O_2_ decreased. This result is consistent with Habash *et al*., who reported that *HsPDI* plays a role in protecting nematodes against ROS^14^, and Dubreuil *et al*., who reported that silencing peroxiredoxin expression in *M*. *incognita* impaired the nematodes infectivity on tomato and their tolerance to exogenous H_2_O_2_^28^.

Previous research of immunolocalization indicated that effector proteins were translocated to the plant apoplast^30^. The *HaEXPB2* from *Heterodera avenae* containing the signal peptide could also target the protein to the apoplast when overexpressed in *N*. *benthamiana*^31^. Our studies of subcellular localization showed that *MgPDI* is localized in apoplast, but how it functioned in the apoplast need to be researched in the further studies.

In conclusion, the combined results from the gene discovery, expression, localization, silencing, and recombinant protein characterization suggest that MgPDI potentially plays an important role in combating the burst of ROS in host plants during the infection process. In brief, MgPDI is a putative virulence factor facilitating *M*. *graminicola* infestation of rice hosts.

## Materials and Methods

### *M*. *graminicola* culture and maintenance

The *M*. *graminicola* isolates ZJJH were maintained on rice (*O*. *sativa* cv. ‘Nipponbare’) grown in potting soil in a growth chamber under a 16 h/8 h light/dark photoperiod at 28 °C/26 °C^32^. Pre-parasitic J2 and parasitic stage nematodes were collected as described by Huang *et al*. and Haegeman *et al*.^32,33^.

### *MgPDI* gene discovery and sequence analysis

Total RNA was extracted from about 10000 freshly hatched second-stage juveniles using the TRIzol reagent (Invitrogen, California, USA). Based on *M*. *graminicola* transcriptome data^34^, the full-length cDNA sequence of *MgPDI* was obtained by rapid amplification of cDNA ends using the SMART RACE cDNA Amplification kit (Clontech, USA) according to the manufacturer’s instructions. PCR-amplified DNA fragments were cloned into pGEM-T Easy Vector (Promega, USA) and then sequenced by Sangon Biotech (Shanghai, China). All primers used in this study were synthesized by Invitrogen Biotechnology Co. Ltd. and are listed in Table 1.

**Table 1:** Primer sequences used in this study.

Primer name	Primer sequences (5′-3′)	Use
MgPDI-3R-F	GTGATGCCACAGCAAACGAT	3′ RACE
MgPDI-3R-R	AAGGCGGTCGTGAAGTGAAT	3′ RACE
MgPDI-F	CTTGTCGAAAATCGATGTTTCG	ORF verification
MgPDI-R	AAGTTTCAGAGTTCATCAGCCG	ORF verification
MgPDI-BamhI	CGCGGATCCATGAGTGATGTCCTTGTATAT	Vector construction
MgPDI-XholI	CCGCTCGAGTCAGAGTTCATCAGCCGGCT	Vector construction
MgPDI-RT-F	TCTCGGCAGAGTATGACGGT	qRT-PCR
MgPDI-RT-R	CTGCGACTTTCTGGAACGAG	qRT-PCR
Mg-ACT-Q-F	AAGATCCTCACTGAGCGTGGTTAC	Actin
Mg-ACT-Q-R	CTTGACCGTCAGGCAATTCATAGC	Actin
MgPDI-P	GAGGGACAGGTTCCGTTTTG	*In situ* hybridization
MgPDI-AP	ACGGTTACGCCAGTAGTTCG	*In situ* hybridization
MgPDI-T7-P	TAATACGACTCACTATAGGGAGGGACAGGTTCCGTTTTG	*In situ* hybridization
MgPDI-T7-AP	TAATACGACTCACTATAGGACGGTTACGCCAGTAGTTCG	*In situ* hybridization
MgPDI-dsRNA-P	GTAAAGCTTATTTTGCCGTC	dsRNA
MgPDI-dsRNA-AP-T7	TAATACGACTCACTATAGGGTCAATGGATTTGTTGCCTGT	dsRNA
MgPDI-dsRNA-P-T7	TAATACGACTCACTATAGGGGTAAAGCTTATTTTGCCGTC	dsRNA
MgPDI-dsRNA-AP	TCAATGGATTTGTTGCCTGT	dsRNA
MgPDI-GFP-P	TCTACAAATCTATCTCTGGATCCATGTTTCGAGAATTATTATA	transient expression
MgPDI -GFP-AP	TCGCCCTTGCTCACCATGGATCCGAGTTCATCAGCCGGCTTCT	transient expression

All primers were 5′ - 3′ orientation.

The deduced protein sequence, pI (isoelectric point) and MW (molecular weight) of MgPDI were predicted by DNAMAN V.6 (Lynnon Biosoft, Que-bec, Canada). An online protein structure homology-modeling server SWISS-MODEL was used to predict the protein tertiary structure. The signal peptide of MgPDI was determined using SignalP 4.1 Server at http://www.cbs.dtu.dk/services/SignalP/ ^35^. Homologous PDI protein sequences from various species were retrieved from the NCBI database and aligned using the program of DNAMAN 6.0.

### *In situ* hybridization

The specific primers PDI-T7-P/PDI-AP and PDI-P/PDI-T7-AP (Table 1) were designed to synthesize DIG-labelled sense and antisense RNA probes (307 bp) using DIG RNA labelling mix (Roche, Basel, Switzerland). *In situ* hybridization was performed according to a modified method of De Boer *et al*. on about 8000 freshly hatched J2s^36^. After hybridization, the probe was detected with an anti-digoxin-FITC monoclonal antibody (diluted 1:1000), and nematodes were imaged using a Laser Scanning Confocal Microscope (Zeiss LSM800, Oberkochen, Germany).

### Developmental expression analysis

Total RNA was extracted from *M*. *graminicola* nematodes at different life stages as described previously^37^ using the TRIzol method (Invitrogen, Carlsbad, CA, USA) according to the manufacturer’s instructions. The cDNA was synthesized using the ReverTra Ace qRT-PCR RT kit (Toyobo, Osaka, Japan). Quantitative RT-PCR was performed with the primer pair MgqRT-PCR-F/MgqRT-PCR-R (Table 1) and actin genes of *M*. *graminicola* were amplified as a reference with the primers Mg-ACT-Q-F/Mg-ACT-Q-R^34^. The qRT-PCR reactions were performed on a CFX Connect Real-Time System (BIO-RAD, USA) using THUNDERBIRD qRT-PCR Mix (Toyobo, Osaka, Japan). Three technical replicates for each reaction were performed and three independent experiments were performed under the following thermal cycler conditions: 95 °C for 60 sec, 40 cycles at 95 °C for 15 sec, and 60 °C for 30 sec. The relative changes in gene expression were determined using the 2^−ΔΔCT^ method^38^.

### Expression, purification, and validation of recombinant MgPDI

The cDNA fragments encoding MgPDI without signal peptide were amplified by PCR primers with restriction sites (Table 1). Products of the PCR assembly were ligated into the pET-32a vector (Novagen, USA) using T4 DNA ligase (Promega, USA). The resulting recombinant vector was validated by PCR product sequencing and was transformed into an *Escherichia coli* BL21 (DE3) competent cell (TaKaRa, China) for protein expression. Transformed *E*. *coli* was cultured at 37 °C and agitated at 180 rpm in lysogeny broth (LB) containing ampicillin (50 μg/ml) until optical density 600 (OD600) reached 0.5. Isopropyl-B-D-thiogalactoside (IPTG) was added to the final concentration of 0.5 mM and then incubated at 20 °C, while shaking at 180 rpm. After incubating for 1 day, the bacterial cells were precipitated by centrifugation and expression analyzed on 12% SDS-PAGE. Thrombin (Sigma, MO, USA) was used to cleave the tags from MgPDI in HisTALON™ Gravity Columns (Clontech, USA) to purify the recombinant proteins, and p-aminobenzamidine-agarose (Sigma, MO, USA) was applied to bind thrombin after cleavage. Lastly, the supernatant was collected and the concentration was measured by absorbance at 280 nm and stored at −80 °C^39^. The purified MgPDI protein band was cut out from the gel after SDS-PAGE and staining by coomassie blue. In-gel proteins were digested overnight in 12.5 ng/mL trypsin in 25 mmol/L NH_4_HCO_3_. The peptide mixtures were injected into the trap column, with a flow rate of 10 μl/min, of a Thermo Scientific Easy nanoLC 1000 (LTQ-Orbitrap Elite, Thermo Fisher Scientific, Waltham, MA, USA) for 2 min to analyze the purified proteins according to the method described by Xu *et al*.^40^.

### PDI activity assays

Isomerization activity of PDI was determined by a standard assay using the modified method previously reported by Hong & Soong^9^. A preparation of 8 μM scrambled RNase A (Sigma) was incubated with 1.4 μM purified recombinant MgPDI protein in a buffer containing 4.5 mM cytidine 2′,3-cyclic monophosphate (cCMP) (Sigma), 1 mM reduced glutathione (Sigma), 0.2 mM oxidized glutathione (Sigma), 2 mM EDTA, and 100 mM Tris–Cl (pH 8.0). The reduction of cCMP by active RNase into CMP was monitored by the absorbance at 296 nm for 60 min in a Microplate Reader (Thermo, Varioskan Flash). For the PDI reductive assay, the MgPDI enzyme activity to reduce insulin was determined by a modified method^41,42^. Reaction mixtures (200 μl) included 100 mM Tris-Cl (pH 6.8), 2 mM ethylenediaminetetraacetic acid (EDTA), 0.13 mM insulin from bovine pancreas (Sigma), 0.33 mM dithiothreitol (DTT) and increasing concentrations (ranging from 2.5 to 10 μM) of purified MgPDI protein. The turbidity of the reaction mixture (Dithiothreitol-mediated insulin reduction by MgPDI) was monitored by measuring the increase of absorbance at 650 nm using a Microplate Reader (Thermo, Varioskan Flash). The reduction of insulin by DTT was recorded in a solution without MgPDI as a negative control.

### Human cell viability assay

The method which assessed cell viability activity of MgPDI on Human Embryonic Kidney 293 (HEK293) cells was modified from the previously described study^43^. The HEK293 cells were adjusted to 3 × 10^4^ cells/well and cultured overnight at 37 °C. Afterwards, the final concentration of MgPDI added to the medium was 0.1, 10 or 1000 ng/ml. After a further 6 h of incubation, H_2_O_2_ was added to a final concentration of 100 μM and cells were incubated for another 30 min in order to induce oxidative stress. Cell viability was measured using a CCK-8 Cell Counting Kit (Vazyme, China) at a wavelength of 450 nm. Cells incubated with 5 μg/ml bovine serum albumin (BSA) were used as a control. Each group was tested in triplicate.

### Protective effect of MgPDI against oxidative damage by an MFO system

A mixed function oxidase (MFO) system was used to generate thiol radicals to damage the plasmid DNA^44^. The MFO system consisting of 1.65 mM DTT, 16.5 mM FeCl_3_ and different concentrations of MgPDI (from 0.1 to 100 μg/mL) were pre-incubated at 37 °C for 2.5 h. Subsequently, pGEM-T Easy plasmid DNA (500 ng, Promega) was added and incubated for 1 h at 37 °C. Nicking of DNA was evaluated by ethidium bromide staining after electrophoresis in 0.8% agarose gels^45^.

### Hydrogen peroxide content determination

The H_2_O_2_ level was determined according to Ji *et al*.^46^. Each sample consisted of approximately 0.1 g fresh roots, collected from knots or tips. Root samples were collected at 3, 7 and 14 dpi, The experiment was performed twice, each time with three replicate samples.

### Double stranded RNA (dsRNA) and infection assay

The forward and reverse primers contained T7 promoter sequences at their 5′ ends for *in vitro* RNA synthesis (Table 1). Double stranded RNA (dsRNA) was synthesized and purified using the T7 RiboMAXTM Express kit (Promega, USA) according to the manufacturer’s instructions. The GFP template was used for the synthesis of a dsRNA construct as a negative control.

RNAi soaking was performed using the modified method of Rosso *et al*. and Huang *et al*.^47,48^. Twenty-five thousand freshly hatched J2s of *M*. *graminicola* were soaked in the dsRNA solution (2 mg mL^−1^ dsRNA, 3 mM spermidine, 50 mM octopamine, and 0.05% gelatin, adjusted with 0.25 × M9 buffer) for 36 h at room temperature in the dark on a rotator. For each reaction, approximately 8000 J2s were used for the plant infection assay; approximately 3000 J2s were used to determine nematode survival after the H_2_O_2_ stress, Freshly hatched J2s of *M*. *graminicola* were soaked in *MgPDI* dsRNA or *GFP* dsRNA (control), dsRNA-treated nematodes were soaked in 20, 40 and 80 mM H_2_O_2_ or in sterile water (0 mM) and live nematodes were counted after 30 min; and all the remaining J2s were used for the qRT-PCR to evaluate the level of *MgPDI* silencing.

For the infection assay, Pluronic F-127 (PF-127) (Sigma-Aldrich) gel was used as previously reported^49,50^. Eighty second-stage juveniles were soaked in dsRNA targeted against *MgPDI* or *GFP* and then inoculated each rice seedling according to Tian^51^. Roots of rice plants were stained with acid fuchsin^52^ and the number of eggs was counted after dissecting the stained galls under a microscope at 15 dpi. To determine the reproductive potential of *M*. *graminicola*, a nematode multiplication factor [MF = (number of egg masses × number of eggs per egg mass) ÷ nematode inoculum level] was calculated.

### Sensitivity of *M*. *graminicola* to hydrogen peroxide (H_2_O_2_)

Approximately 200 freshly hatched J2s were incubated in each treatment of 0, 10, 20, 40 or 80 mM of H_2_O_2_. Dead nematodes were counted after 30, 60, 90 and 120 min and the percentage of survival was calculated. Treatments were replicated three times and the experiment was repeated three times. To determine *MgPDI* gene expression in individuals under H_2_O_2_ stress, 5000 freshly hatched J2s were incubated in each treatment of 20, 40 or 80 mM H_2_O_2_ for 30 min, and washed in sterile tap water. Individuals of J2s incubated in sterile tap water were used as the control. The relative expression levels were quantified in H_2_O_2_-incubated nematodes relative to J2s soaked in sterile distilled water (0 mM H_2_O_2_) by qRT-PCR as described above.

### Subcellular localization of *MgPDI*

The coding sequences of *MgPDI* was cloned into the pGD-eGFP vector, and the *MgPDI*:: pGD-eGFP construct was transformed into the *Agrobacterium tumefaciens* strain EHA 105. The method of *Agrobacterium*-mediated transient expression was carried out as previously described^14,31^. The *A*. *tumefaciens* suspensions were adjusted with the infiltration buffer to an OD600 of 1.0 and mixed with an RNA-silencing suppressor P19 at 1:1. After incubation for 2 h at RT, the *A*. *tumefaciens* strains carrying the constructs were injected in the leaves abaxial side of 6 week-old *Nicotiana benthamiana* plant using 1 mL hypodermic syringe without needle. To distinguish the cell wall and the plasma membrane, the leaf cells were plasmolysed by infiltrating 1 M NaCl_2_ solution for 10 min before observation. Infiltrated plants were incubated in the growth chamber (16 hrs light, 8 hrs dark and at 25 °C) for 5 days^31^. The primers sequences are given in Table 1.

## Supplementary information

supplementary file

Supplementary Table S1

## Data Availability

All data generated or analysed during this study are included in this published article (and its Supplementary Information files).
