# Supplementary material for: Meloidogyne graminicola protein disulfide isomerase may be a nematode effector and is involved in protection against oxidative damage
Source: Sci Rep. 2019 Aug 16;9:11949. doi: 10.1038/s41598-019-48474-w (PMC6697734; doi:10.1038/s41598-019-48474-w)
Supplement: Supplementary file 1 — supplementary file [file 41598_2019_48474_MOESM1_ESM.pdf]

Supplementary Information for

Meloidogyne graminicola protein disulfide isomerase may be a nematode effector and is involved in protection against oxidative damage

Zhong-ling Tian [1], Ze-hua Wang [2], Munawar Maria [1] , Nan Qu [1], Jing-wu Zheng [1, 3]*

[1]Laboratory of Plant Nematology, Institute of Biotechnology, College of Agriculture and Biotechnology, Zhejiang University, Hangzhou 310058, Zhejiang, P.R. China.

[2]Institute of Insect Science, College of Agriculture and Biotechnology, Zhejiang University, Hangzhou 310058, Zhejiang, P.R. China.

[3]Key Lab of Molecular Biology of Crop Pathogens and Insects, Ministry of Agriculture, Hangzhou 310058, P.R.China

Email addresses:

Zhong-ling Tian: tzl@zju.edu.cn

Ze-hua Wang: 382732003@zju.edu.cn

Munawar Maria: maria.munawar@yahoo.com

Nan Qu: 1397483265@qq.com

Jing-wu Zheng: jwzheng@zju.edu.cn

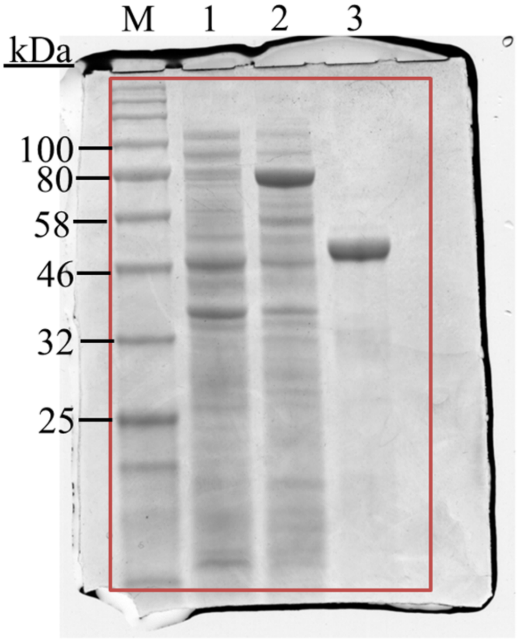

Supplementary Figure S1. The full gel of Figure 3. The original gel of Coomassie brilliant blue G-250 staining. Lanes: 1, the total proteins; 2, the expressed product of pET-32a-MgPDI induced by IPTG; 3, enriched nontagged MgPDI. SDS-PAGE image was acquired using Densitometer GS-900 (Bio-rad, Hercules, CA, USA).

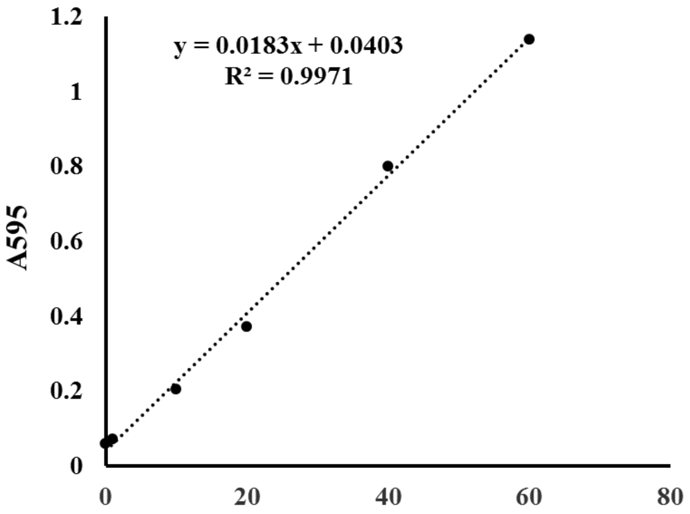

Supplementary Figure S2. Standard curve for hydrogen peroxide content determination
